# B cell pathway dual inhibition for systemic lupus erythematosus: a prospective single‐arm cohort study of telitacicept

**DOI:** 10.1002/mco2.515

**Published:** 2024-03-23

**Authors:** Lanlan Ji, Yan Geng, Xiaohui Zhang, Xuerong Deng, Zhibo Song, Meng Tan, Ying Tan, Chenxue Qu, Zhuoli Zhang

**Affiliations:** ^1^ Department of Rheumatology and Clinical Immunology Peking University First Hospital Beijing China; ^2^ National Clinical Research Center for Skin and Immune Diseases Beijing China; ^3^ Department of Nephrology Peking University First Hospital Beijing China; ^4^ Department of Laboratory Medicine Peking University First Hospital Beijing China

**Keywords:** B cell inhibitor, transmembrane activator, calcium modulator and cyclophilin ligand interactor (TACI), lupus, telitacicept, B lymphocyte stimulator (BLyS), a proliferation‐inducing ligand (APRIL)

## Abstract

Systemic lupus erythematosus (SLE) is a heterogeneous autoimmune disease associated with B‐cell hyperactivity. Telitacicept is a transmembrane activator, calcium modulator, and cyclophilin ligand interactor‐Fc fusion protein, which can neutralize both B‐cell lymphocyte stimulator and a proliferation‐inducing ligand. Patients with active SLE who received telitacicept were prospectively followed at month 1, 3, 6, 9, and 12 after telitacicept initiation. Thirty‐seven participants were involved and followed for 6.00 [3.00, 6.00] months. SRI‐4 rate at month 6 was 44.7%. The median dosage of prednisone was decreased by 43.8% (from 10 to 5.62 mg/d) at month 6. The anti‐dsDNA level was significantly decreased, while complement levels were significantly increased at month 6 from baseline. Continuously significant reductions in serum immunoglobin (Ig)G IgA, and IgM levels were also observed. Patients experienced significant decreases in the numbers of total and naive B cells, whereas memory B cells and T cell populations did not change. The number of NK cells was significantly increased during the follow‐up. At month 6, 58.3% (14 out of 24) patients experienced improved fatigue accessed by FACIT–Fatigue score exceeding the minimum clinically important difference of 4. Most adverse events were mild, but one each case of severe hypogammaglobulinemia, psychosis with suicidal behavior, and B‐cell lymphoma were occurred. In our first prospective real‐world study, telitacicept treatment led to a significant clinical and laboratory improvement of disease activity, as well as fatigue amelioration in patients with SLE. Safety profile was favorable overall, but more studies are greatly needed.

## Graphical abstract

Telitacicept is a novel fusion protein constructed with the extracellular domain of the human transmembrane activator and calcium modulator and cyclophilin ligand interactor (TACI) receptor and the fragment crystallizable (Fc) domain of human immunoglobulin G (IgG). TACI can bind either B lymphocyte stimulator (BLyS) or another TNF family ligand known as a proliferation‐inducing ligand (APRIL), which are prominent factors in B cell differentiation. Telitacicept can prevent BlyS and APRIL from binding to BlyS receptor 3 (BR3, also known as BAFF‐R), TACI and B cell maturation antigen receptors expressed on the B‐cell surface, inhibiting the development and survival of mature B cells and plasma cells.

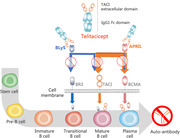

## 1 INTRODUCTION

Systemic lupus erythematosus (SLE), a chronic autoimmune disease, often leads to multisystem inflammation and organ injury. Hydroxychloroquine, glucocorticoids, and immunosuppressants have been the mainstay of therapy. Although the survival of patients with SLE has been greatly improved during the past decades, the mortality is still approximately threefold higher than the general population.^1^ The leading causes of death have been reported as infection, renal failure, and cerebrovascular diseases.^1^ The incidence and prevalence of SLE vary in different races, more common in African Americans, Hispanics, and Asians than in Caucasians.^2^ Current therapy strategies, which mainly rely on high dose of glucocorticoids and immunosuppressants, are efficacious only in certain patients. Residual disease activity or severe side effects of medications usually cause organ damage or even life‐threatening consequences. So, management of SLE and its diverse clinical manifestations remain challengeable. There is great unmet need in the novel targeted therapies for SLE patients.^3^

Although inheritance, hormone, and environment have been identified as risk factors of developing SLE,^4^, ^5^ abnormal activation of immune system is the core mechanism characterized by exaggerated B cell responses and production of autoantibodies.^2^ The B‐cell lymphocyte stimulator (BLyS; also known as the B‐cell activation factor [BAFF]) and a proliferation‐inducing ligand (APRIL) are involved in B‐cell survival and differentiation. BLyS modulates the differentiation and maturation of immature B cell, while APRIL modulates the function and survival of long‐lived plasma cell.^6^ BLyS and APRIL were found to be increased in SLE, and thus they might play prominent roles in the pathogenesis of SLE.^7^, ^8^ Three receptors of BLyS and APRIL have been identified as transmembrane activator and calcium modulator cyclophilin ligand interactor (TACI), B cell maturation antigen (BCMA) and BAFF receptor (BAFF‐R). BAFF‐R has a strong selectivity for BLyS; BCMA has a higher affinity for APRIL than for BLyS, while TACI binds both ligands equally well.^9^

Telitacicept is a novel, recombinant fusion protein, consisting of TACI and the Fc portion of human immunoglobin G (IgG) (TACI‐Ig).^10^ It is anticipated to interfere with abnormal B cell and plasma cell activation by antagonizing the interaction of BLyS and APRIL with their respective receptors on the surface of B lymphocytes. Evidence suggests that TACI‐Ig could reduce the increased serum level of BLyS and thereby inhibit subsequent activation of B cell‐driven mechanisms.^11^ Based on the previous efficacy and safety data of phase IIb trial, telitacicept was approved in China for the treatment of adult SLE patients with inadequate responses to standard‐of‐care (SoC) in March 2021. Additionally, the U.S. Food and Drug Administration has granted fast track designation to telitacicept for the treatment of SLE in April 2020.^10^, ^12^ The phase IIb data were just published a few days ago but phase III data have not been published yet.^13^ Moreover, few real‐world data of telitacicept use and patient outcomes have been reported.

Therefore, we investigated the clinical effectiveness, fatigue, and safety outcomes of telitacicept plus SoC in two prospective cohorts of SLE patients over 12 months in clinical practice. Moreover, the present study focused more on the effects of telitacicept treatment on biomarkers, including serological levels of Ig, complements, select B and T cell populations, and biomarkers as predictors of treatment response. We aimed to provide more detailed and comprehensive evidence and facilitate the further application of telitacicept in SLE.

## 2 RESULTS

### 2.1 Patient characteristics

The enrollment flow chart is shown in Figure S1. In total, 37 patients contributing 161 clinic visits were included. The baseline characteristics of the 37 patients are presented in Table 1. At the enrollment, the median age was 34.6 [28.9, 46.1] with median disease duration of 9.48 [2.94, 16.3] years. 97.3% of them were with serologically active (defined as anti‐dsDNA positive and/or hypocomplementemia) SLE. The median follow‐up was 6.00 [3.00, 6.00] months.

**TABLE 1:** Demographics and clinical characteristics of 37 SLE patients.

Characteristics of patients		Cohort (*n* = 37)
Demographics	Female, *n* (%)	33.0 (89.2)
	Age at enrollment, y	34.6 [28.8, 46.1]
	Age at disease onset, y	24.0 [20.0, 30.0]
Clinical manifestations	Disease duration, y	9.48 [2.94, 16.3]
	Manifestations, *n* (%)	
	Constitutional	1.00 (2.70)
	Cutaneous	16.0 (43.2)
	Musculoskeletal	3.00 (8.11)
	Serositis	2.00 (5.41)
	Hematological	
	Leucopenia	6.00 (16.2)
	Autoimmune hemolytic anemia	0.00 (0.00)
	Thrombocytopenia	4.00 (10.8)
	Lupus nephritis	17.0 (45.9)
	Neuropsychiatric	1.00 (2.70)
	Cardiovascular	2.00 (5.41)
	Antiphospholipid antibody with consecutive positivity, n (%)	11.0 (29.7)
	Serologically active, *n* (%)	
	Anti‐dsDNA positive	31.0 (83.8)
	Hypocomplementemia	27.0 (73.0)
	SLE disease activity index (SLEDAI)	8.00 [5.00, 10.0]
	<4, *n* (%)	2.00 (5.40)
	>=4, *n* (%)	35.0 (94.6)
	Physician global assessment (PGA)	1.00 [1.00, 1.50]
	System damage index (SDI) category, *n* (%)	
	0	21.0 (56.8)
	≥1	16.0 (43.2)
Treatment	Prednisone daily dosage or equivalent, mg	10.0 [5.00, 20.0]
	Hydroxychloroquine, *n* (%)	32.0 (86.5)
	Immunosuppressant use, *n* (%)	30.0 (81.1)
	Mycophenolate mofetil, *n* (%)	23.0 (62.2)
	Cyclophosphamide, *n* (%)	2.00 (5.40)
	Tacrolimus, *n* (%)	2.00 (5.40)
	Cyclosporin, *n* (%)	2.00 (5.40)
	Azathioprine, *n* (%)	1.00 (2.70)
	Regimen of telitacicept, *n* (%)	
	80 mg per week	5.00 (13.5)
	160 mg per week	32.0 (86.5)
	Previous exposure to biologics within 3 months before initiating telitacicept, *n* (%)	4.00 (10.8)
	Belimumab	3.00 (8.11)
	Rituximab	1.00 (2.70)

Therapeutically, the median concomitant prednisone daily dosage or equivalent dosage of other glucocorticoids was 10.0 [5.00, 20.0] mg. The dosage was less than 15 mg/day in most patients [26 out of 37 (70.3%)], followed by more than 30 mg [six out of 37 (16.2%)] and 15−30 mg [five out of 37 (13.5%)].

### 2.2 Efficacy outcomes

**FIGURE 1:**
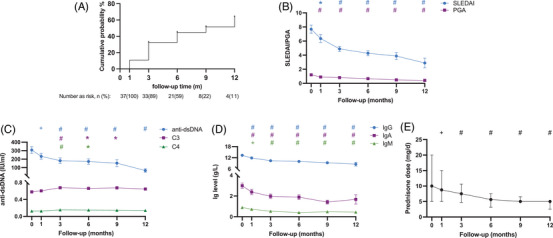
Effects of telitacicept treatment on disease activity, autoantibody, complement, immunoglobulin levels, and prednisone dosage. (A) Kaplan–Meier curve with cumulative probability of SRI‐4 response. (B) Changes in SLEDAI and PGA of all patients. (C) Changes in the levels of anti‐double‐stranded DNA (anti‐dsDNA) and complement (including C3 and C4) of all patients. (D) Changes in the levels of IgG, IgA, and IgM. (E) Changes in prednisone daily dosage. The *p* values were shown with different colors according to the corresponding lines. * = *p* < 0.05; + = *p* < 0.01; # = *p* < 0.001 versus baseline. The figure of prednisone dose was shown as median with IQR, the others were shown as mean with standard error of mean.

During the follow‐up, 18 out of 37 (48.7%) patients reached SRI‐4. The Kaplan–Meier estimate of the probability of SRI‐4 response after Telitacicept initiation is presented in Figure 1A. The cumulative probability of SRI‐4 response was 10.8% at 1 months, 32.4% at 3 months, 44.7% at 6 months, 51.6% at 9 months, and 63.7% at 12 months. As one of the components of SRI‐4, the mean SLEDAI was declined significantly over time, from 7.68 ± 3.50 at baseline to 4.27 ± 2.00 at 6 months (*p* < 0.001), and 2.89 ± 2.09 at 12 months (*p* < 0.001) (Figure 1B). As for BILAG, all 37 patients (100%) achieved no new BILAG A organ domain score and no more than 1 new B organ domain score. The mean PGA score was declined from 1.21 ± 0.44 at baseline to 0.66 ± 0.45 at 6 months (*p* < 0.001), and 0.42 ± 0.50 at 12 months (*p* < 0.001) (Figure 1B).

There were 6 flares accessed by SFI during the follow‐up. The risk of flare was 6.06% (two out of 33), 8.33% (three out of 36), 3.33% (one out of 30), 0% (zero out of 16) and 0% (zero out of 9) for month 1, 3, 6, 9, and 12, respectively. Among them, there was only one severe flare [2.78% (one out of 36)] at month 3.

The effects of telitacicept on serologic biomarkers are presented in Figure 1C. Of note, telitacicept resulted in significantly lower anti‐dsDNA levels compared with baseline as early as month 1, and this trend of reduction was sustained through month 12 (mean reduction of 24.1, 40.6, 43.9, 50.2, and 78.6% at month 1, 3, 6, 9, and 12, respectively, *p* < 0.001 for month 3–12). We also found the significantly increased C3 and C4 levels during the follow‐up, as early as month 3 comparing with baseline (mean increase of 17.0 and 22.2% at month 3, 14.2 and 19.0% at month 6, 16.6 and 15.1% at month 9, 11.6 and 11.1% at months 12, for C3 and C4 respectively, *p* < 0.001 for month 3 and 6).

Following telitacicept treatment, the serum levels of Ig, including IgG, IgA, and IgM, were all significantly decreased. The significant decrease of Ig levels can be observed as early as month 1 and at each visit thereafter. At month 6, the mean drop in IgG, IgA, and IgM level was 3.28 g/L (21.8%), 1.10 g/L (36.7%), and 0.49 g/L (54.3%), respectively, *p* < 0.001 for all; at month 12, the mean decrease in IgG, IgA, and IgM level was 4.76 g/L (35.8%), 1.31 g/L (43.9%), and 0.45 g/L (49.8%), respectively, *p* < 0.001 for all (Figure 1D).

The dosage of glucocorticoids was significantly lowered during the follow‐up. The median reduction of prednisone daily dosage was 1.25 mg (12.5%) at month 1 (*p* = 0.001), 2.50 mg (25.0%) at month 3, 4.38 mg (43.8%) at month 6, 5.00 mg (50.0%) at month 9 and 12, respectively, *p* < 0.001 for month 3–12 (Figure 1E).

### 2.3 Subgroup analyses in patients with a stable SoC regimen

There were 24 patients with stable dosage and types of glucocorticoids as well as immunosuppressants for at least 3 months before initiating telitacicept. During the follow‐up, 10 out of 24 (41.7%) patients receiving telitacicept reached SRI‐4. The cumulative probability of SRI‐4 response after telitacicept initiation was 8.30% at month 1, 29.2% at month 3, 38.6% at month 6 and 9, 69.3% at month 12. We still found the significantly decreased mean SLEDAI score over time, from 7.12 ± 2.76 at baseline to 4.35 ± 2.18 at month 6, and 2.20 ± 1.48 at month 12 (*p* < 0.001) (Figure S2A). Meanwhile, the mean PGA score was also declined from 1.17 ± 0.42 at baseline to 0.70 ± 0.51 at month 6, and 0.30 ± 0.52 at month 12 (*p* < 0.001) (Figure S2A). For the serologic biomarkers, we only observed the significant improvement for both anti‐dsDNA and complement level at month 3 (Figure S2B), not the subsequent time points. Like the whole group, the serum levels of immunoglobulins, including IgG, IgA, and IgM, were also significantly decreased over time (Figure S2C). After telitacicept treatment, the median prednisone daily dosage was reduced to from 6.88 [3.44, 10.0] mg/d at baseline to 5.00 [2.50, 6.25] at month 6, and 3.75 [2.50, 5.00] mg/d at month 12 (*p* < 0.001) (Figure S2D).

### 2.4 Effect of telitacicept on lymphocyte subpopulations

**FIGURE 2:**
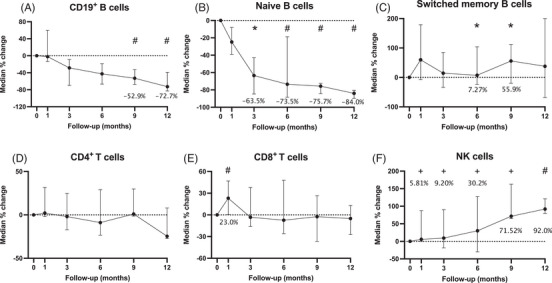
Changes in lymphocyte subsets numbers from baseline to 9 months after telitacicept initiation. (A) CD19^+^B cells; (B) naive B cells; (C) switched memory B cells; (D) CD4^+^ T cells; (E) CD8^+^ T cells; (F) NK cells. Numbers represented graphically are the actual percentages. * = *p* < 0.05; + = *p* < 0.01; # = *p* < 0.001. The figures were shown as median with IQR.

There were 35, 18, 32, 27, 11, and 8 patients with available data of lymphocytes subsets analysis at baseline, month 1, 3, 6, 9, and 12, respectively. In general, telitacicept treatment significantly reduced median levels of total B cells throughout 12 months (−2.14 to −72.7%, from month 1 to 12, *p* < 0.001), while preserving memory B cell populations. Median reductions in CD19^+^ B cells were only statistically significant at month 9 and month 12 comparing with baseline, while significant reductions in naïve B (CD20^+^CD38^−^CD27^−^IgD^+^) cells were observed from month 3 to 9 (Figures 2A and B). In contrast, there was a trend of increase of switched memory B cells (CD20^+^CD38^−^CD27^+^IgD^−^), only statistically significant at month 6 and 9 (59.9 to 37.9%, from month 1 to 12, *p* = 0.07) (Figure 2C). Decreased plasmablast (−57.6%) and plasma cells (−81.7%) could also be found at month 9 compared with baseline; however, statistically significant decrease was only in plasma cells (*p* < 0.001) (Figure S3).

We did not observe significant change in the median CD3^+^CD4^+^ or CD3^+^CD8^+^ T cell counts from baseline during the 12‐month treatment with telitacicept, except for a modest (23.0%) expansion of CD3^+^CD8^+^ cells at month 1 (Figures 2D and E). The median number of NK cells was increased steadily during the treatment, with statistical significance at all the follow‐up visits comparing with baseline (*p* < 0.001) (Figure 2F).

### 2.5 The predictive factors of SRI‐4 response

Several baseline variables were identified to be potentially correlated with SRI‐4 response, including skin and renal involvement, urine examinations, anti‐U1RNP, anti‐Sm, SLEDAI and PGA in univariate Cox analysis. Further multivariate analysis indicated that anti‐U1RNP and SLEDAI were independent predictive factors [anti‐U1RNP: hazard ratio [HR] 3.67 (1.10, 12.2), *p* = 0.04; SLEDAI: HR 1.55 (1.24, 1.93), *p* < 0.001] (Table 2).

**TABLE 2:** Baseline predictors of SRI‐4 response by univariate and multivariate Cox analysis.

	Univariate	*p*	Multivariate	*p*
Skin involvement	3.48 (1.26, 9.61)	0.016	1.85 (0.57, 6.05)	0.306
Lupus nephritis	4.11 (1.42, 11.9)	0.009	1.28 (0.34, 4.76)	0.716
Urine dipstick protein	1.70 (1.09, 2.65)	0.020	/	/
24‐h urine protein	1.22 (1.08, 1.39)	0.002	/	/
Urine active sediment	3.11 (1.12, 8.59)	0.029	/	/
Anti‐U1RNP	3.44 (1.20, 9.91)	0.022	3.67 (1.10, 12.2)	0.035
SLEDAI	1.57 (1.32, 1.87)	<0.001	1.55 (1.24, 1.93)	<0.001
PGA	6.17 (1.99, 19.1)	0.002	/	/

The variables included in the univariate analysis were multiple demographics, clinical, laboratory, and treatment variables. Only the variables with *p* value < 0.03 were shown in the table. There was strong collinearity between lupus nephritis and urine examinations, anti‐U1RNP, anti‐Sm, as well as SLEDAI and PGA. So only lupus nephritis, anti‐U1RNP, and SLEDAI were included in the multivariate analysis.

Among the changes of clinical variables from baseline, only the change in IgA level at month 1 [HR 0.95 (95% confidence interval [CI] 0.91, 0.99), *p* = 0.01], and the change in SLEDAI at month 3 [HR 0.98 (95% CI 0.96, 0.99), *p* = 0.03] were the independent predictive factors of SRI‐4 achievement during the follow‐up (Table 3).

**TABLE 3:** Predictors at month 1 and 3 of SRI‐4 response by univariate and multivariate Cox analysis.

	Changes from baseline (%)	Univariate	*p*	Multivariate model 1	*p*	Multivariate model 2	*p*
At month 1	C3	1.02 (0.99, 1.04)	0.065	0.99 (0.97, 1.02)	0.548	/	/
	IgG	0.95 (0.91, 0.99)	0.013	/	/	/	/
	IgA	0.95 (0.93, 0.98)	<0.001	0.95 (0.91, 0.99)	0.008	/	/
	PGA	0.99 (0.97, 0.99)	0.049	0.98 (0.96, 1.00)	0.128	/	/
At month 3	C3	1.02 (1.00, 1.03)	0.007	/	/	1.00 (0.99, 1.02)	0.346
	C4	1.00 (1.00, 1.02)	0.011	/	/	/	/
	IgM	0.97 (0.95, 1.00)	0.051	/	/	/	/
	IgA	0.98 (0.96, 0.99)	0.020	/	/	0.98 (0.97, 1.01)	0.153
	PGA	0.98 (0.97, 0.99)	0.011	/	/	/	/
	SLEDAI	0.97 (0.95, 0.99)	0.001	/	/	0.98 (0.96, 0.99)	0.017

The variables included in the univariate analysis were the percentage changes in anti‐dsDNA, C3, C4, IgG, IgA, IgM levels, SLEDAI, and PGA from baseline to month 1 and 3. Only the variables with *p* value < 0.07 were shown in the table. There might be collinearity between C3 and C4 levels, IgG, IgA, and IgM levels, as well as SLEDAI and PGA. So only the change in C3 level, IgA and SLEDAI were included in the multivariate analysis if they were coexisted.

Among the variables of lymphocytes subsets at baseline and the changes of these variables from baseline to month 1 and 3 in percentage, only the change of NK cell count at month 3 showed independent predictive value to SRI‐4 achievement, however with low HR value [HR 1.003 (95% CI 1.000, 1.006), *p* = 0.04] (Table S1).

### 2.6 Fatigue outcome

**FIGURE 3:**
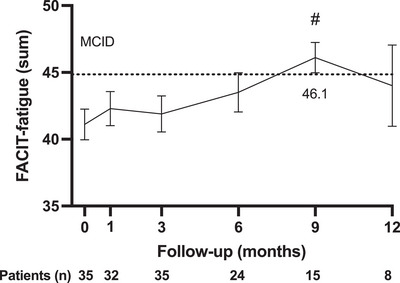
The mean changes of FACIT‐Fatigue score from baseline through 12 months after telitacicept initiation. The dotted line showed the level of MCID comparing with baseline. Numbers represented graphically are the actual sum score of FACIT‐Fatigue. # = *p* < 0.001. The figure was shown as mean with standard error of mean.

The FACIT–Fatigue score was significantly increased at month 9 from baseline (46.1 ± 4.43 vs. 41.1 ± 6.80, *p* < 0.001) (Figure 3). The patients experienced an improvement in the FACIT‐Fatigue score that exceeded the minimum clinically important difference (MCID) (≥4) were 25.0% (eight out of 32), 25.7% (nine out of 35), 58.3% (14 out of 24), 53.3% (eight out of 15) and 50.0% (four out of eight) at month 1, 3, 6, 9, and 12, respectively.

### 2.7 Safety outcomes

During treatment, no patients died. Three (8.11%) serious AE were reported, with severe hypogammaglobulinemia (IgG < 5 g/L) accompanied by hospitalization due to posterior circulation encephalopathy in one patient, serious depression with suicide behavior in one patient, and diffuse large B‐cell lymphoma in one patient. Telitacicept was discontinued in all of them. Psychiatric events were reported in two patients. Except for the patient with suicide behavior, another patient with psychosis had a history of anxiety before. Among the infections, no serious infection was observed. More than half of the infections were COVID‐19, during the outbreak in China December 2022. The percentage of COVID‐19 was 40.5% (15 out of 37) and 12.9% (16 out of 124) for patient and person‐visit level respectively. Other infections were upper respiratory infection in most patients [13.5% (five out of 37)], followed by urinary tract infection and herpes zoster [2.70% (one out of 37)]. Among other adverse events (AEs), injection site reactions included local pruritus or pain which resolved within 2−3 days (Table 4).

**TABLE 4:** Percentage of adverse events in patient level and person‐time level (excluding COVID‐19).

	Patients (*n* = 37)	Person‐visit (*n* = 124)
Total AE, *n* (%)	67.6% (25/37)	35.5% (44/124)
Serious AE, *n* (%)	8.11% (3/37)	2.42% (3/124)
Severe hypogammaglobulinemia and hospitalization	2.70% (1/37)	0.81% (1/124)
Depression with suicide behavior	2.70% (1/37)	0.81% (1/124)
Tumor	2.70% (1/37)	0.81% (1/124)
Infections except COVID‐19, *n* (%)	18.9% (7/37)	7.26% (9/124)
Severe infection	0	0
Upper respiratory infection	13.5% (5/37)	5.65% (7/124)
Urinary tract infection	2.70% (1/37)	0.81% (1/124)
Herpes zoster	2.70% (1/37)	0.81% (1/124)
Injection site reactions, *n* (%)	18.9% (7/37)	13.7% (17/124)
Psychiatric events, *n* (%)	5.40% (2/37)	1.61% (2/124)
Tumor, *n* (%)	2.70% (1/37)	0.81% (1/124)
AE resulting in reduction or discontinuation of telitacicept, *n* (%)	8.11% (3/37)	2.42% (3/124)

## 3 DISCUSSION

In this prospective real‐world study, we demonstrated 44.7% of SRI‐4 achievement after telitacicept treatment in addition to SoC for 6 months. The clinical improvement was accompanied by decreased glucocorticoid daily dosage and immunoglobin levels. Importantly, for the first time, we evaluated the impact of telitacicept on lymphocytes populations and fatigue in SLE patients.

SRI‐4, as a composite assessment of SLE response index, was firstly used in the clinical trials of Belimumab. In those belimumab randomized control studies (RCTs), the SRI‐4 response rate at week 52 varied between 43.2 and 58%.^14^, ^15^ Although not a head‐to‐head comparison between belimumab and telitacicept, telitacicept seemed to have a higher SRI‐4 rate, which varied between 68.3 and 82.6% (conference abstract).^13^, ^16^ Other noncontrolled studies of telitacicept demonstrated a similar SRI‐4 rate between 66.7 and 80.9%.^17^, ^18^, ^19^ But the baseline glucocorticoid dosage of these previous studies was much higher than ours. The therapeutic effect of newly increased or added glucocorticoids could not be excluded. Importantly, in our subgroup analysis of the patients with a stable SoC regimen for at least 3 months, the SRI‐4 achievement was 41.7%, like those RCT data of belimumab. To be noted that there were 2 patients with SLEDAI < 4 at baseline, suggesting the SRI‐4 response rate might be underestimated in our study. Overall, the results of our study indicated that the efficacy of telitacicept was at least comparable to belimumab in the treatment of SLE.

Recognizing the predictors of SRI‐4 response is valuable for clinical practice. Although the predictors may vary depending on the study design, population, interventions and many others, there are some baseline factors that have been identified and proved by several studies, such as higher disease activity and active serological markers.^20^, ^21^ We also confirmed higher SLEDAI at baseline as the independent predictor of SRI‐4 response. Among the early predictors after treatment, Parodis et al.’s^22^ study found that early expansion of memory B cells at week 8 was the predictor of SRI‐4 response at week 52. More prominent rapid reductions in anti‐dsDNA and increases in complement levels at week 8 were also documented in SRI‐4 responders.^22^ The only independent predictor of SRI‐4 achievement among lymphocyte subsets in our study was the increase of NK cells at month 3; however, the HR value was close to 1 unfortunately. The other early independent predictors were the changes of IgA at month 1 and the SLEDAI at month 3 after treatment.

Telitacicept was designed to inhibit the activity of both BAFF and APRIL. It has been anticipated to interfere with abnormal activation of B cells and plasma cells. Nevertheless, the effect of inhibition on B cell subsets and plasma cells by telitacicept has not been reported yet. Our results for the first time demonstrated that telitacicept could significantly reduce the number of total B and naive B cells, while preserving memory B cell populations and T cells in lupus patients. These results were consistent with that of Belimumab, although we did not find significantly increased memory B cells at the initial phase of treatment.^23^ The ranges of plasmablast and plasma cell change were much wider than the other types of B cells. It was likely due to the relatively small proportions of these subsets in the overall B cell population, leading to a higher variability in count. Despite nonsignificantly decreased plasma cell count, the levels of immunoglobins, the product of plasma cells, were significantly decreased after initiation of telitacicept. Presumably the function of over‐activated plasma cells was inhibited. Another striking finding was the steadily increased NK cells during telitacicept treatment. It is not known yet whether BAFF/APRIL inhibition influences proliferation of NK cells, resulting in increased NK cell count. But several studies have reported the negative correlation of circulating NK cell count with disease activity in SLE,^24^, ^25^ which might be a mechanism attributable to its beneficial effect in SLE.

Overall, the safety profile of telitacicept in our study was similar to previous reports. The percentage of infections (excluding COVID‐19) in our study was lower than those in the other studies.^18^, ^26^ The unexpected COVID‐19 outbreak in China did not increase severe infections during telitacicept treatment. Another fusion protein that blocks BAFF and APRIL in the treatment of SLE is atacicept. Although atacicept 150 mg group suggested benefit in efficacy versus placebo in a RCT (APRIL‐SLE), this study arm was terminated prematurely due to two deaths caused by infection.^27^ It seemed that the different molecular constitution of telitacicept from atacicept had made a favorable safety profile of infection. The phase 2 trial in IgA nephropathy revealed significantly decreased serum immunoglobulin levels.^26^ There was one patient discontinued telitacicept due to severe hypogammaglobulinemia in our study. Although the concomitant use of high dose prednisone and cyclophosphamide might also contribute to hypogammaglobulinemia in this patient, the risk of hypogammaglobulinemia should be closely monitored in the future. Psychosis was reported in two patients and importantly one had suicidal behavior. The Medicines and Healthcare products Regulatory Agency ever released a warning of increased risk of serious psychiatric events in SLE patients receiving belimumab in 2019.^28^ Although a meta‐analysis of RCTs showed belimumab therapy overall does not increase psychiatric events,^29^ our real‐world data again illuminate a red flag warning of close monitoring for the patients receiving belimumab or telitacicept. History of psychosis should also be considered before telitacicept initiation. It was noteworthy that one patient developed lymphoma in our study. The association between tumor and B cell inhibition treatment had not been established. However, patients with B‐cell mediated diseases, such as Sjögren's syndrome and SLE, were reported to have increased risks of developing B‐cell lymphoma.^30^ So, we think the development of B‐cell lymphoma in this patient was probably due to lupus instead of telitacicept.

We acknowledge several limitations of this study. First, relatively small sample size due to the newly approval of telitacicept for clinical use and COVID‐19 pandemic restrictions. Further studies involving more patients with control groups should be considered to confirm our results. Second, SDI and other life quality measurements were not evaluated due to limited time of follow‐up. Third, all the enrolled patients were Chinese, therefore the results might primarily represent in this patient group. To be noted that a global multicenter phase III clinical trial was approved by the European Union and the National Medicines Administration in September 2022. Ethnicity diversity in the global trial would facilitate the generalizability of telitacicept in broader population. Finally, different education level of the patients might be a confounding factor to influence the accuracy of FACIT questionnaire.

In conclusion, there was superior clinical efficacy with favorable safety profile of telitacicept in the treatment of SLE. The clinical improvement was accompanied by decreased steroid dose, immunoglobin levels, total and naive B cell subsets. Telitacicept could also improve the fatigue. Further studies with more patients and longer observation period are critically warranted.

## 4 MATERIALS AND METHODS

The study was reported according to the Strengthening the Reporting of Observational Studies in Epidemiology statement.

### 4.1 Study design and participants

In this study, the patients were enrolled from two lupus cohorts running in Peking University First Hospital. The STAR (treat SLE to TARget) cohort, established in 2007, is a prospective longitudinal observational cohort of rheumatology department. The STAR cohort has been described in greater detail in our previous researches.^31^, ^32^, ^33^ All patients fulfilled either the 1997 revised SLE ACR classification criteria or the 2012 SLICC classification criteria. The PKULN (PeKing University Lupus Nephritis) cohort is a prospective, longitudinal observational cohort of nephrology department. All patients in this cohort had biopsy proved lupus nephritis class II to V defined by International Society of Nephrology and Renal Pathology Society within 3 months before at the enrollment.

This was a single‐center, observational, single‐armed study. Patients in STAR and PKULN cohorts fulling the following criteria from May 19, 2022 to April 3, 2023 were consecutively included in this study: (1) age ≥ 18 years; (2) SLE disease activity index 2000 (SLEDAI‐2k) ≥ 4 or 1 ≤ SLEDAI‐2k < 4 plus British Isles lupus assessment group index (BILAG) more than one category of A or B^34^, ^35^; and (3) Telitacicept was initiated in addition to SoC by the decision of the treating rheumatologist, with the aim of either controlling disease activity or reducing glucocorticoid dose. Patients with less than 3 months follow‐up data were excluded (Figure S1). Ethics approval was obtained from the ethics committee of the Peking University First Hospital (project 2022[194]). Written informed consent was obtained from all participants.

### 4.2 Clinical assessments and data collection

Demographics, disease duration, SLE core set variables, laboratory findings, and treatment details were documented at baseline. SLEDAI‐2k score, BILAG index, and physician's global assessment (PGA) of disease activity on a 0−3 cm visual analog scale, anchored at 0 (none) and 3 (severe), with intermediate lines at 1 (mild) and 2 (moderate) were also evaluated.^36^ Fatigue was assessed with the FACIT–Fatigue scale, a 13‐item scale, that measures physical and mental fatigue and their effects on functioning and daily living. Scores range from 0 to 52, with lower scores indicating more fatigue.^37^, ^38^, ^39^, ^40^ After that, all patients are prospectively followed up, evaluated, and documented at 1, 3, 6, 9, and 12 months. Changes in B cell subsets, T cells (CD3^+^CD4^+^ and CD3^+^CD8^+^) and NK (CD16^+^CD56^+^) cells were analyzed in a subgroup of patients. B cell subsets consisted of total B (CD19^+^), transitional (CD20^+^CD38^+^CD27^−^CD24^+^), naïve (CD20^+^CD38^−^CD27^−^IgD^+^), unswitched memory (CD20^+^CD38^−^CD27^+^IgD^+^), and switched memory (CD20^+^CD38^−^CD27^+^IgD^−^) subsets. Plasma cell subsets consisted of plasmablast (CD38^+^CD27^+^CD20^−^CD138^−^) and plasma cells (CD38^+^CD27^+^CD20^−^CD138^+^). The flowcytometry was performed by using BD FACS Canto2 in the central laboratory of Peking University First Hospital.

### 4.3 Outcomes

The efficacy outcome was 6‐month response rate as assessed by the SLE responder index 4 (SRI‐4). SRI‐4 is defined as a 4‐point improvement in the SLEDAI−2k score, with no new BILAG A organ domain score and no more than 1 new B organ domain score, and no worsening (0.3‐point increase) in the PGA score.^41^ The occurrence of flare, defined by the SELENA‐SLEDAI flare index (SFI) as mild/moderate flare or severe flare, was also recorded at each assessment point.^42^ The changes of glucocorticoid dosage, serological parameters, immunoglobin levels and lymphocytes populations were evaluated. As for the fatigue outcome, a MCID of 4 units was used for assessment of the FACIT–Fatigue scores. All the AEs were collected and reported.

### 4.4 Statistical analysis

Patient characteristics were presented as mean (S.D.) or median [interquartile range (IQR)] depending on the level of resemblance to the normal distribution. Absolute and relative frequencies were reported for categorical variables. The trends of glucocorticoids dose, disease and serological activity, as well as lymphocytes subsets during the study period were analyzed using generalized estimating equations with an unstructured working correlation matrix and a robust estimation for the covariance matrix. SRI‐4 response rates were measured by aforementioned definitions, using Kaplan–Meier analysis and reported as percentages with 95% CI. The Cox proportional hazards model was used to identify predictors of SRI‐4 response. To determine the independent predictors, factors with a *p* value < 0.07 at univariate analysis were then entered into the multivariate model. P‐values were presented with the HR and corresponding 95% CI in the Cox model. Analyses were performed with R version 4.2.1 (R Foundation for Statistical Computing, Vienna, Austria). GraphPad Prism V.9.0 were used to produce the graphs. The level of significance was set at a two‐sided *p* value < 0.05.

## AUTHOR CONTRIBUTIONS

Z. Z. was responsible for the study design, participated in its design and coordination, and critically revised the manuscript. L. J. had full access to all the data collection, analysis, interpretation, and drafted the manuscript. Y. G., X. Z., X. D., Z. S., M. T., and Y. T. contributed to the process of data collection. C. Q. contributed to the flow assessment of lymphocytes. All authors have read and approved the final manuscript.

## CONFLICT OF INTEREST STATEMENT

The authors declared no conflict of interest.

## ETHICS STATEMENT

This study was approved by the Ethics Committee of Peking University First Hospital (IRB number 2022[194]). Written informed consent was obtained from all participants.

## Supporting information

Supporting Information

## Data Availability

All data relevant to the study are included in the article or uploaded as Supplemental information.

## References

[1] Mu L , Hao Y , Fan Y , et al. Mortality and prognostic factors in Chinese patients with systemic lupus erythematosus. Lupus. 2018;27(10):1742‐1752. doi:10.1177/0961203318789788 30060721

[2] Kiriakidou M , Ching CL . Systemic lupus erythematosus. Ann Intern Med. 2020;172(11):ITC81‐ITC96. doi:10.7326/AITC202006020 32479157

[3] Tsokos GC . Systemic lupus erythematosus. N Engl J Med. 2011;365(22):2110‐2121. doi:10.1056/NEJMra1100359 22129255

[4] Cooper GS , Dooley MA , Treadwell EL , St Clair EW , Parks CG , Gilkeson GS . Hormonal, environmental, and infectious risk factors for developing systemic lupus erythematosus. Arthritis Rheum. 1998;41(10):1714‐1724. doi:10.1002/1529-0131(199810)41:10<1714::AID‐ART3>3.0.CO;2‐U 9778212

[5] Lahita RG . The role of sex hormones in systemic lupus erythematosus. Curr Opin Rheumatol. 1999;11(5):352‐356. doi:10.1097/00002281-199909000-00005 10503654

[6] Shi F , Xue R , Zhou X , Shen P , Wang S , Yang Y . Telitacicept as a BLyS/APRIL dual inhibitor for autoimmune disease. Immunopharmacol Immunotoxicol. 2021;43(6):666‐673. doi:10.1080/08923973.2021.1973493 34519594

[7] Vincent FB , Morand EF , Schneider P , Mackay F . The BAFF/APRIL system in SLE pathogenesis. Nat Rev Rheumatol. 2014;10(6):365‐373. doi:10.1038/nrrheum.2014.33 24614588

[8] Nakayamada S , Tanaka Y . BAFF‐ and APRIL‐targeted therapy in systemic autoimmune diseases. Inflamm Regen. 2016;36:6. doi:10.1186/s41232-016-0015-4 29259679 PMC5725651

[9] Bossen C , Schneider P . BAFF, APRIL and their receptors: structure, function and signaling. Semin Immunol. 2006;18(5):263‐275. doi:10.1016/j.smim.2006.04.006 16914324

[10] Fan Y , Gao D , Zhang Z . Telitacicept, a novel humanized, recombinant TACI‐Fc fusion protein, for the treatment of systemic lupus erythematosus. Drugs Today Barc Spain 1998. 2022;58(1):23‐32. doi:10.1358/dot.2022.58.1.3352743 35107091

[11] Liu Y , Zhang L , Wu Y , et al. Therapeutic effects of TACI‐Ig on collagen‐induced arthritis by regulating T and B lymphocytes function in DBA/1 mice. Eur J Pharmacol. 2011;654(3):304‐314. doi:10.1016/j.ejphar.2011.01.002 21244850

[12] Dhillon S . Telitacicept: first approval. Drugs. 2021;81(14):1671‐1675. doi:10.1007/s40265-021-01591-1 34463932

[13] Wu D , Li J , Xu D , et al. Telitacicept in patients with active systemic lupus erythematosus: results of a phase 2b, randomised, double‐blind, placebo‐controlled trial. *Ann Rheum Dis* . Published online December 21, 2023. doi:10.1136/ard-2023-224854 PMC10958275 38129117

[14] Navarra SV , Guzmán RM , Gallacher AE , et al. Efficacy and safety of belimumab in patients with active systemic lupus erythematosus: a randomised, placebo‐controlled, phase 3 trial. Lancet Lond Engl. 2011;377(9767):721‐731. doi:10.1016/S0140-6736(10)61354-2 21296403

[15] Furie R , Petri M , Zamani O , et al. A phase III, randomized, placebo‐controlled study of belimumab, a monoclonal antibody that inhibits B lymphocyte stimulator, in patients with systemic lupus erythematosus. Arthritis Rheum. 2011;63(12):3918‐3930. doi:10.1002/art.30613 22127708 PMC5007058

[16] Telitacicept, a Human Recombinant Fusion Protein Targeting B Lymphocyte Stimulator (BlyS) and a Proliferation‐Inducing Ligand (APRIL), in Systemic Lupus Erythematosus (SLE): Results of a Phase 3 Study. ACR Meeting Abstracts. Accessed May 29, 2023. https://acrabstracts.org/abstract/telitacicept‐a‐human‐recombinant‐fusion‐protein‐targeting‐b‐lymphocyte‐stimulator‐blys‐and‐a‐proliferation‐inducing‐ligand‐april‐in‐systemic‐lupus‐erythematosus‐sle‐results‐of‐a‐phase‐3‐study/

[17] Sun L , Shen Q , Gong Y , et al. Safety and efficacy of telitacicept in refractory childhood‐onset systemic lupus erythematosus: a self‐controlled before‐after trial. Lupus. 2022;31(8):998‐1006. doi:10.1177/09612033221097812 35499216

[18] Chen R , Fu R , Lin Z , Huang C , Huang W . The efficacy and safety of telitacicept for the treatment of systemic lupus erythematosus: a real life observational study. Lupus. 2023;32(1):94‐100. doi:10.1177/09612033221141253 36416639

[19] Jin HZ , Li Y‐J , Wang X , et al. Efficacy and safety of telitacicept in patients with systemic lupus erythematosus: a multicentre, retrospective, real‐world study. Lupus Sci Med. 2023;10(2):e001074. doi:10.1136/lupus-2023-001074 38007228 PMC10679987

[20] van Vollenhoven RF , Petri MA , Cervera R , et al. Belimumab in the treatment of systemic lupus erythematosus: high disease activity predictors of response. Ann Rheum Dis. 2012;71(8):1343‐1349. doi:10.1136/annrheumdis-2011-200937 22337213 PMC3396451

[21] Gatto M , Saccon F , Zen M , et al. Early disease and low baseline damage as predictors of response to belimumab in patients with systemic lupus erythematosus in a real‐life setting. Arthritis Rheumatol. 2020;72(8):1314‐1324. doi:10.1002/art.41253 32275125

[22] Parodis I , Gomez A , Lindblom J , Chow JW , Doria A , Gatto M . Early changes in B and plasma cell subsets and traditional serological markers as predictors of sri‐4 response to therapy in systemic lupus erythematosus. Front Med. 2022;9:852162. doi:10.3389/fmed.2022.852162 PMC9096349 35572992

[23] Stohl W , Hiepe F , Latinis KM , et al. Belimumab reduces autoantibodies, normalizes low complement levels, and reduces select B cell populations in patients with systemic lupus erythematosus. Arthritis Rheum. 2012;64(7):2328‐2337. doi:10.1002/art.34400 22275291 PMC3350827

[24] Henriques A , Teixeira L , Inês L , et al. NK cells dysfunction in systemic lupus erythematosus: relation to disease activity. Clin Rheumatol. 2013;32(6):805‐813. doi:10.1007/s10067-013-2176-8 23377197

[25] Spada R , Rojas JM , Barber DF . Recent findings on the role of natural killer cells in the pathogenesis of systemic lupus erythematosus. J Leukoc Biol. 2015;98(4):479‐487. doi:10.1189/jlb.4RU0315-081RR 26216938

[26] Lv J , Liu L , Hao C , et al. Randomized phase 2 trial of telitacicept in patients with IgA nephropathy with persistent proteinuria. Kidney Int Rep. 2023;8(3):499‐506. doi:10.1016/j.ekir.2022.12.014 36938094 PMC10014376

[27] Isenberg D , Gordon C , Licu D , Copt S , Rossi CP , Wofsy D . Efficacy and safety of atacicept for prevention of flares in patients with moderate‐to‐severe systemic lupus erythematosus (SLE): 52‐week data (APRIL‐SLE randomised trial). Ann Rheum Dis. 2015;74(11):2006‐2015. doi:10.1136/annrheumdis-2013-205067 24951103 PMC4680140

[28] Belimumab (Benlysta▼): increased risk of serious psychiatric events seen in clinical trials. GOV.UK. Accessed July 8, 2023. https://www.gov.uk/drug‐safety‐update/belimumab‐benlysta‐increased‐risk‐of‐serious‐psychiatric‐events‐seen‐in‐clinical‐trials

[29] Xie W , Huang H , Zhan S , Zhang Z . Risk of psychiatric disorders and all‐cause mortality with belimumab therapy in patients with systemic lupus erythematosus: a meta‐analysis of randomised controlled trials. Lupus Sci Med. 2021;8(1):e000534. doi:10.1136/lupus-2021-000534 34697129 PMC8547509

[30] Mörth C , Valachis A , Abu Sabaa A , et al. Autoimmune disease in patients with diffuse large B‐cell lymphoma: occurrence and impact on outcome. Acta Oncol Stockh Swed. 2019;58(8):1170‐1177. doi:10.1080/0284186X.2019.1619936 31131659

[31] Gao D , Hao Y , Mu L , et al. Frequencies and predictors of the lupus low disease activity state and remission in treatment‐naïve patients with systemic lupus erythematosus. Rheumatol Oxf Engl. 2020;59(11):3400‐3407. doi:10.1093/rheumatology/keaa120 32337549

[32] Hao Y , Oon S , Ji L , et al. Determinants and protective associations of the lupus low disease activity state in a prospective Chinese cohort. Clin Rheumatol. 2022;41(2):357‐366. doi:10.1007/s10067-021-05940-z 34595670 PMC8782788

[33] Ji L , Gao D , Hao Y , et al. Low‐dose glucocorticoids withdrawn in systemic lupus erythematosus: a desirable and attainable goal. Rheumatol Oxf Engl. 2022;62(1):181‐189. doi:10.1093/rheumatology/keac225 35412598

[34] Gladman DD , Ibañez D , Urowitz MB . Systemic lupus erythematosus disease activity index 2000. J Rheumatol. 2002;29(2):288‐291. 11838846

[35] Hay EM , Bacon PA , Gordon C , et al. The BILAG index: a reliable and valid instrument for measuring clinical disease activity in systemic lupus erythematosus. Q J Med. 1993;86(7):447‐458. 8210301

[36] Aranow C , Askanase A , Oon S , et al. Laboratory investigation results influence Physician's Global Assessment (PGA) of disease activity in SLE. Ann Rheum Dis. 2020;79(6):787‐792. doi:10.1136/annrheumdis-2019-216753 32241797

[37] Yellen SB , Cella DF , Webster K , Blendowski C , Kaplan E . Measuring fatigue and other anemia‐related symptoms with the Functional Assessment of Cancer Therapy (FACT) measurement system. J Pain Symptom Manage. 1997;13(2):63‐74. doi:10.1016/s0885-3924(96)00274-6 9095563

[38] Webster K , Cella D , Yost K . The Functional Assessment of Chronic Illness Therapy (FACIT) measurement system: properties, applications, and interpretation. Health Qual Life Outcomes. 2003;1:79. doi:10.1186/1477-7525-1-79 14678568 PMC317391

[39] Kosinski M , Gajria K , Fernandes AW , Cella D . Qualitative validation of the FACIT‐fatigue scale in systemic lupus erythematosus. Lupus. 2013;22(5):422‐430. doi:10.1177/0961203313476360 23423250

[40] Strand V , Berry P , Lin X , Asukai Y , Punwaney R , Ramachandran S . Long‐Term impact of belimumab on health‐related quality of life and fatigue in patients with systemic lupus erythematosus: six years of treatment. Arthritis Care Res. 2019;71(6):829‐838. doi:10.1002/acr.23788 PMC6593666 30320964

[41] Furie RA , Petri MA , Wallace DJ , et al. Novel evidence‐based systemic lupus erythematosus responder index. Arthritis Rheum. 2009;61(9):1143‐1151. doi:10.1002/art.24698 19714615 PMC2748175

[42] Petri M , Buyon J , Kim M . Classification and definition of major flares in SLE clinical trials. Lupus. 1999;8(8):685‐691. doi:10.1191/096120399680411281 10568907

