# Supplementary material for: B cell pathway dual inhibition for systemic lupus erythematosus: a prospective single‐arm cohort study of telitacicept
Source: MedComm (2020). 2024 Mar 23;5(4):e515. doi: 10.1002/mco2.515 (PMC10960726; doi:10.1002/mco2.515)
Supplement: Supplementary file 1 — Supporting Information [file MCO2-5-e515-s001.docx]

**B cell pathway dual inhibition for systemic lupus erythematosus: a prospective single-arm cohort study of telitacicept**

**Running title**: Telitacicept in lupus

Lanlan Ji^1,2^, Yan Geng^1,2^, Xiaohui Zhang^1,2^, Xuerong Deng^1,2^, Zhibo Song^1,2^, Meng Tan^3^, Ying Tan^3^, Chenxue Qu^4^, Zhuoli Zhang^1,2^

^1^Department of Rheumatology and Clinical Immunology, Peking University First Hospital, Beijing, China

^2^National Clinical Research Center for Skin and Immune Diseases, China

^3^Department of Nephrology, Peking University First Hospital, Beijing, China

^4^Department of Laboratory Medicine, Peking University First Hospital, Beijing, China

**Corresponding author:** Zhuoli Zhang, [zhuoli.zhang@126.com](mailto:zhuoli.zhang@126.com), Director, Rheumatology and Clinical Immunology Department, Peking University First Hospital, No.8, Xishiku Street West District Beijing 100034 China

**Acknowledgments:** None

**SUPPLEMENTARY APPENDIX**

**Supplementary figure S1.** The enrollment flow chart

**Supplementary figure S2.** Effects of telitacicept treatment on disease activity, autoantibody, complement, immunoglobulin levels and glucocorticoids dosage in the patients with stable treatment regimen within 3 months before the initiation of talitecicept.

**Supplementary figure S3.** Changes in other B cell subsets counts from baseline to 9 months after telitacicept initiation.

**Supplementary table S1.** Lymphocyte subsets predictors of SRI4 response by univariate and multivariate Cox analysis

**Supplementary figure S1.** The enrollment flow chart

**Supplementary figure S2**. Effects of telitacicept treatment on disease activity, autoantibody, complement, immunoglobulin levels and glucocorticoids dosage in the patients with stable treatment regimen within 3 months before the initiation of talitecicept. A, Changes in SLEDAI and PGA. B, Changes in the levels of anti–double-stranded DNA (anti-dsDNA) and complement (including C3 and C4). C, Changes in the levels of IgG, IgA and IgM. D, Changes in prednisone daily dosage. The p value were shown with different colors according to the corresponding lines. *= P<0.05; += P<0.01; #=P<0.001 versus baseline. The figure of glucocorticoids dose was shown as median with IQR, the others were shown as mean with standard error of mean.

**Supplementary figure S3.** Changes in some B cell subsets from baseline to 9 months after telitacicept initiation. A, Change in transitional B cells counts; B, Change in unswitched memory B cells counts; C, Change in plasmablast cells counts; D, Change in plasma cells counts. Numbers of actual percentages are represented graphically.*=p<0.05; #=p<0.001. The figures were shown as median with IQR.

**Supplementary table S1.** Lymphocyte subsets predictors of SRI4 response by univariate and multivariate Cox analysis

|  |  | Univariate | p | Multivariate Model | p |
| --- | --- | --- | --- | --- | --- |
| baseline | NK cell number | 0.993 (0.986, 1.000) | 0.053 | 0.998 (0.990, 1.006) | 0.706 |
| month 3 | NK cell change % | 1.004 (1.001, 1.007) | 0.002 | 1.003 (1.000, 1.006) | 0.043 |
|  | CD8^+^ T cell change % | 1.006 (0.999, 1.012) | 0.051 | 1.004 (0.997, 1.011) | 0.235 |

The variables included in the univariate analysis were lymphocyte subsets counts at baseline and the percentage changes of these variables from baseline to month 1 and 3. Only the variables with p value<0.06 were shown in the table. Data were shown as HR (95% CI).
